# Aqueous synthesis of highly adsorptive copper–gallic acid metal–organic framework

**DOI:** 10.1038/s41598-020-75927-4

**Published:** 2020-11-05

**Authors:** Badril Azhar, Artik Elisa Angkawijaya, Shella Permatasari Santoso, Chintya Gunarto, Aning Ayucitra, Alchris Woo Go, Phuong Lan Tran-Nguyen, Suryadi Ismadji, Yi-Hsu Ju

**Affiliations:** 1 Department of Chemical Engineering, National Taiwan University of Science and Technology, #43, Sec. 4, Keelung Rd., Taipei, 106 Taiwan; 2 Graduate Institute of Applied Science and Technology, National Taiwan University of Science and Technology, #43, Sec. 4, Keelung Rd., Taipei, 106 Taiwan; 3 Department of Chemical Engineering, Widya Mandala Surabaya Catholic University, Kalijudan 37, Surabaya, 60133 Indonesia; 4 Department of Mechanical Engineering, Can Tho University, Campus II, 3/2 street, Can Tho city, 900100 Vietnam; 5 Taiwan Building Technology Center, National Taiwan University of Science and Technology, #43, Sec. 4, Keelung Rd., Taipei, 106 Taiwan

**Keywords:** Environmental impact, Chemical engineering, Materials science

## Abstract

A greener route to synthesize mesoporous copper–gallic acid metal–organic framework (CuGA MOF) than the conventional method using harmful DMF solvent was proposed in this study. Various synthesis attempts were conducted by modifying the synthesis conditions to produce CuGA MOF with comparable physical properties to a reference material (DMF-synthesized CuGA NMOF). The independent variables investigated include the molar ratio of NaOH to GA (1.1 to 4.4) and the synthesis temperature (30, 60, 90 °C). It was found that proper NaOH addition was crucial for suppressing the generation of copper oxide while maximizing the formation of CuGA MOF. On the other hand, the reaction temperature mainly affected the stability and adsorption potential of CuGA MOF. Reacting Cu, GA, and NaOH at a molar ratio of 1:1:2.2 and a temperature of 90 °C, produced mesoporous MOF (CuGA **90–2.2**) with a surface area of 198.22 m^2^/g, a pore diameter of 8.6 nm, and a thermal stability of 219 °C. This MOF exhibited an excellent adsorption capacity for the removal of methylene blue (124.64 mg/g) and congo red (344.54 mg/g). The potential usage of CuGA **90–2.2** as a reusable adsorbent was demonstrated by its high adsorption efficiency (> 90%) after 5 adsorption–desorption cycles.

## Introduction

The ever-growing population and developing industries cause major impact on the environment. Water emerges as one of the highly affected resources due to the increasing usage of synthetic dyes in various sectors, including food processing, pharmaceutical, cosmetic, plastic and textile industries^1^. Annually, over 7∙10^5^ tons of synthetic dyes have been generated and approximately 10–15% of it was leached out during coloring process and may end up in the water bodies. Anionic dyes (i.e., congo red, CR) and cationic dyes (i.e., methylene blue, MB) are the most frequent synthetic dyes found in dye-contaminated water^2,3^. Earlier studies have reported the adverse effect of these dyes for environment and human health, where extended contact may trigger kidney disease, cancer and allergies^4,5^. Due to the severity of dye contamination, several removal methods, such as electrolysis, membrane filtration, photo-assisted degradation, and adsorption, have been developed^6–8^. Among these methods, adsorption is a favorable approach due to its low energy consumption and processing cost, safer by-product, and simple regeneration techniques^9,10^. Therefore, it triggers researches on the development of various adsorbent material with efficient dye removal ability.

In the past decades, metal–organic frameworks (MOFs) have emerged as one of the extensively studied adsorbents. MOFs offer better adsorptive performance than the commonly used adsorbent such as bentonite, activated carbon, and zeolite^11–14^. This superiority is attributed to their high surface area, large pore volume, and numerous adsorption sites^15^. Copper-gallic acid MOF (CuGA MOF) is a bio-MOF since it composes biologically active phytochemical (i.e., GA) and essential metal element (i.e., Cu)^16,17^. To date, the applicability of CuGA MOF is inferior to other extensively studied MOFs such as MIL-100, MIL-101, UiO-66, ZIF-8^18^. While the application of CuGA MOFs as drug carrier with superior antitumor activity has been reported previously^19^, their adsorptive behavior remains elusive. The preparation of CuGA MOF was first introduced by Sharma et al. who reacted Cu(II) and GA with a mixture of solvents containing Aerosol OT, *n*-butanol, and *N*,*N*-dimethylformamide (DMF)^19^. However, the use of DMF is not desirable due to its harmful potential to the environment^20^. Hence, it is necessary to develop a greener route for synthesizing CuGA.

In this work, the aqueous synthetic method involving the use of NaOH is explored. NaOH was used to create alkaline condition to trigger the dissociation of organic ligands making it accessible for interaction with the metal core^16,21,22^. The optimum molar ratio of NaOH to GA which is a prerequisite for the formation of CuGA MOF was determined. This optimum ratio restricts the formation of undesirable product, such as metal hydroxide and oxide species, which were commonly found in NaOH-rich condition^22,23^. Concurrently, the developed synthesis method eliminates the use of organic solvent and reduces the reaction time to 2 h. In addition, the effect of synthesis temperature on the adsorption ability of the CuGA MOFs was highlighted and the reusability of CuGA MOF for the removal of CR and MB was assessed in this study.

## Results and discussion

In the following sections, the synthesized CuGA MOF samples were designated as CuGA ***T-X***; with ***T*** refers to the synthesis temperature, and ***X*** refers to the molar ratio of NaOH to GA.

### Effect of NaOH to GA molar ratio on the characteristics of CuGA

**Figure 1:**
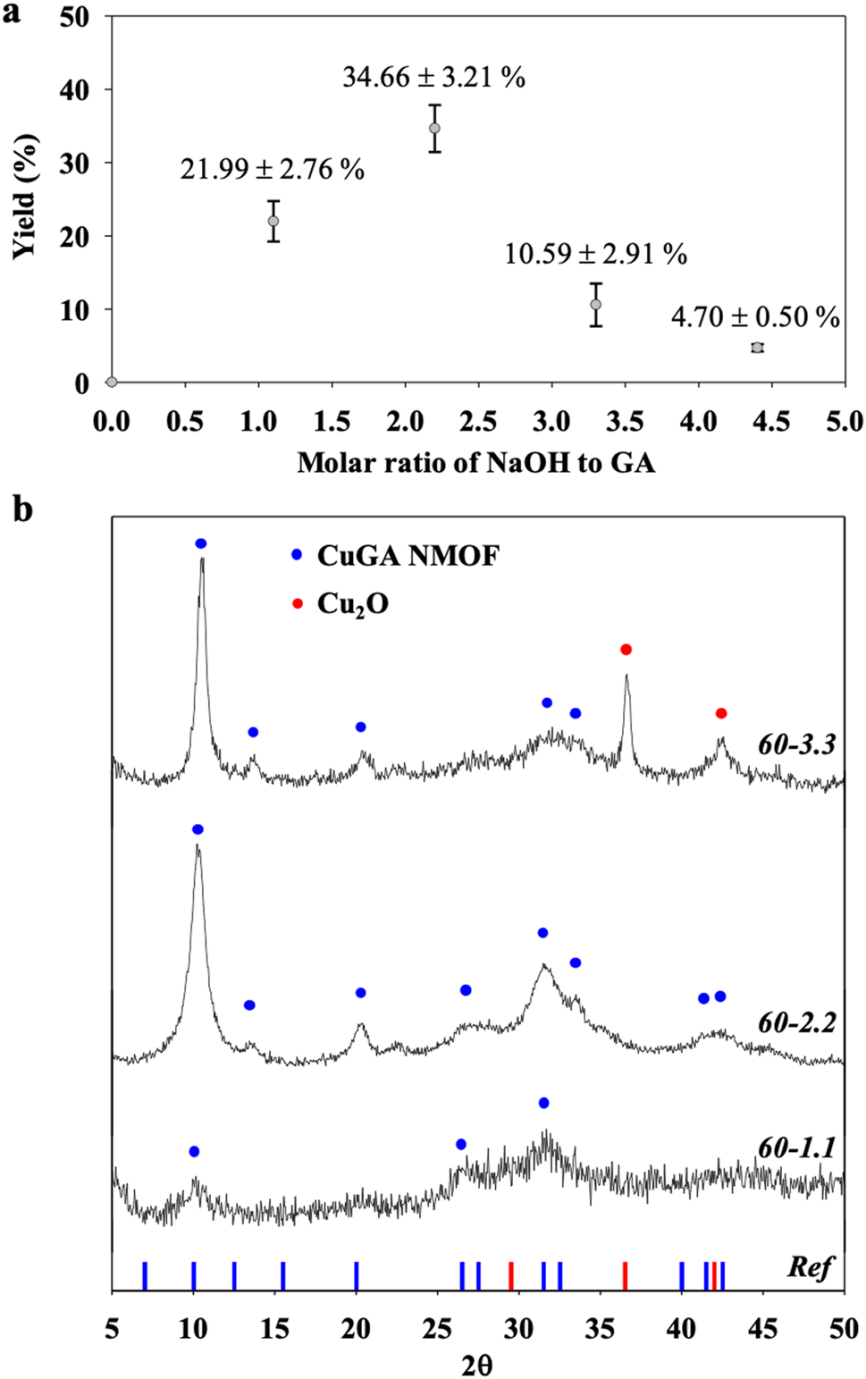
(**a**) Yield and (**b**) PXRD of CuGA synthesized at 60 °C with different molar ratio of NaOH to GA. The reference XRD pattern was adopted from reported data by Sharma et al.^19^.

To obtain the optimum conditions for CuGA MOFs synthesis, reactions were carried out at different reaction temperatures and molar ratios of the reactants. The first set of reactions were done by varying ***X ***with temperature fixed at 60 °C. The purpose was to determine the proper amount of NaOH required to produce CuGA MOF with comparable characteristics to that of the reference material, CuGA NMOF^19^. Product yield and XRD pattern were employed as the basis for this screening. As shown in Fig. 1a, the highest yield (34.66%) was obtained at ***X*** = 2.2.

A comparison of the PXRD patterns between CuGA **60-X** and reference CuGA NMOF is given in Fig. 1b. The closest resemblance to the CuGA NMOF PXRD pattern was observed in the CuGA **60–2.2**, specifically with the occurrence of peaks at 2θ = 10.1, 13.2, 20.1, 28.0, 31.1, and 42.8°. The PXRD pattern of CuGA NMOF was also quite prominent for CuGA **60–3.3**, albeit the inclusion of Cu_2_O characteristic peaks at 2θ = 36.56° and 42.39°. The formation of Cu_2_O can be attributed to the oxidation activity that occurs due to the presence of excessive NaOH^24^. These results suggest that ***X*** = 2.2 is the most suitable NaOH to GA molar ratio for the synthesis of CuGA MOF, and therefore, this NaOH concentration was chosen for further studies. The amount of NaOH involved in the synthesis also significantly affected the crystallinity of the CuGA. Based on the PXRD pattern analysis, it was observed that CuGA **60–1.1** exhibited lower crystallinity (23.9%) than that of CuGA **60–2.2** (40.1%) and CuGA **60–3.3** (35.4%). These results illustrate the importance of NaOH in the formation of CuGA. NaOH has a modulatory role in promoting the coordination between Cu and GA. As a base, NaOH triggers the deprotonation of carboxylic acid and hydroxyl group of GA, thus produces electron-rich GA. This negatively-charged GA then provides better attraction towards the surrounding metal ions and facilitates interaction of Cu and GA^19^. At a lower NaOH concentration (***X*** = 1.1), there was only a limited amount of OH^−^ ions available for GA deprotonation. Therefore, the affinity of GA molecules toward the metal ions was low. On the other hand, at a higher NaOH concentration (***X*** = 3.3 and 4.4), excessive OH^−^ ions may inhibit the Cu to GA coordination bonds and triggers the formation of metal hydroxide compound instead^22^.

### Effect of temperature on the synthesis of CuGA

**Figure 2:**
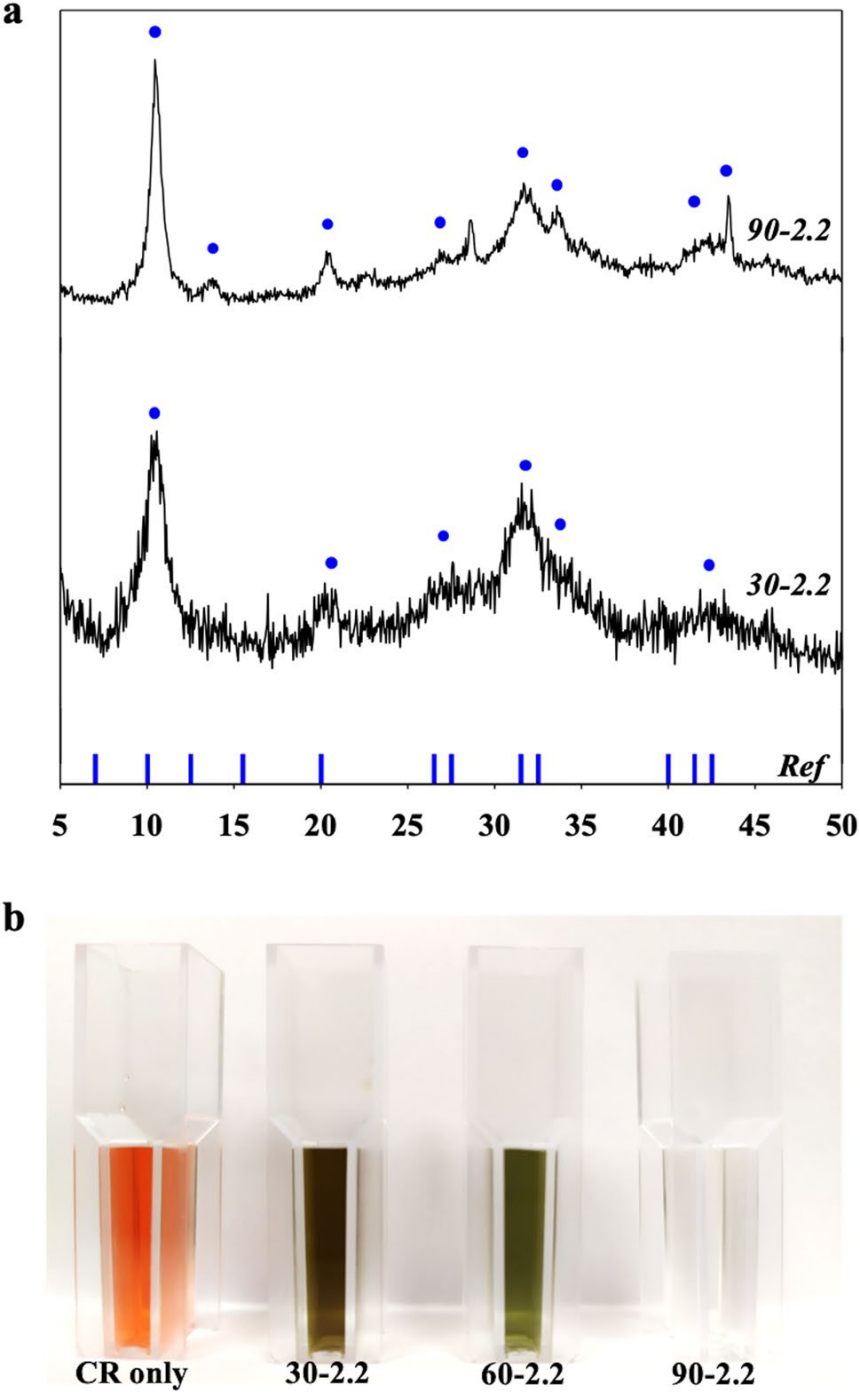
(**a**) PXRD of CuGA synthesized at different synthesis temperatures The reference XRD was based on the reported CuGA NMOF^19^. (**b**) Adsorption ability of CuGA **T-2.2** for removal of 1000 ppm CR dye.

To evaluate the effect of temperature on the characteristic of CuGA, ***X*** was set at 2.2 while synthesis temperatures of 30, 60, and 90 °C were used to produce CuGA **30–2.2**, CuGA **60–2.2**, and CuGA **90–2.2**, respectively. As presented in Fig. 2a, the PXRD patterns for CuGA **30**–**2.2** and CuGA **90**–**2.2** samples are comparable to CuGA **60**–**2.2** (Fig. 1b) and the reference CuGA NMOF. While temperature may not significantly affect the crystal patterns of the CuGA MOFs, it did significantly affect their adsorption ability (Fig. 2b). After 24 h of incubation, CuGA MOF **90–2.2** showed significant removal of CR, as justified by the transparent supernatant. Meanwhile, brownish-green-colored supernatants were formed in the systems containing CuGA **30–2.2** and CuGA **60–2.2**. This aberrant discoloration can be attributed to the presence of radical species that were generated from the MOF degradation which sequentially triggered chromogenic reaction of CR^25^. This result suggests that higher synthesis temperature is preferred to form more stable coordination between Cu and GA.

### Characterization of CuGA MOF

**Figure 3:**
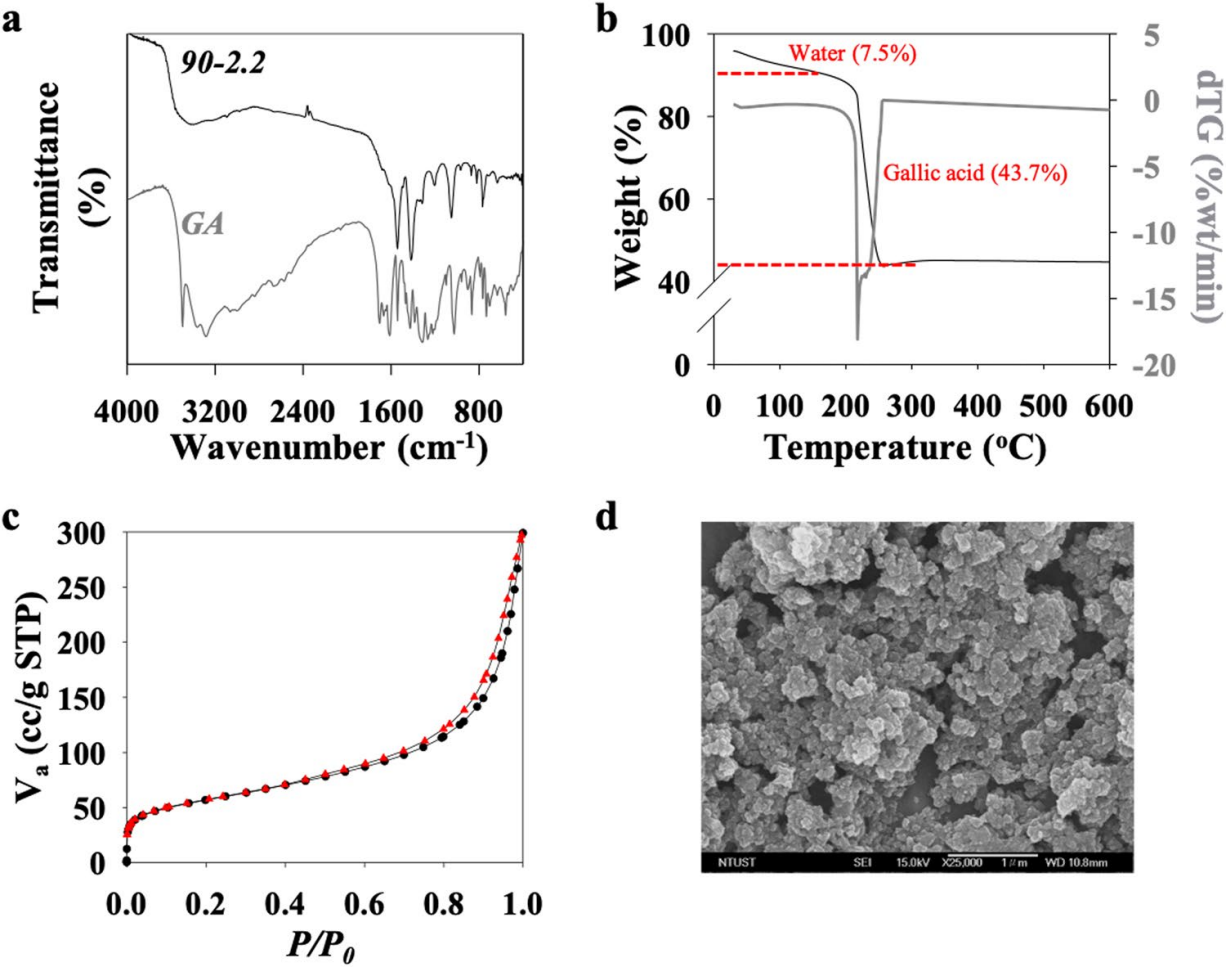
(**a**) FTIR chromatogram, (**b**) TGA thermogram, (**c**) N_2_ sorption isotherm, and **(d)** SEM image of CuGA **90–2.2**.

As described above, CuGA **90–2.2** showed similar PXRD pattern to that of the reference material and exhibited excellent adsorption potential. Thus, this MOF was further characterized. The comparison between the FTIR spectra of GA and CuGA **90–2.2** is shown in Fig. 3a. It was observed that the bands correspond to the vibration of the hydroxyl (–OH) groups at 3494, 3338, 3263 cm^−1^ and the vibration of the carbonyl (C=O) group at 1699 cm^−1^ disappear in the CuGA **90–2.2** chromatogram, confirming the participation of the –OH and the C=O groups in the formation of CuGA. In addition, several band shifts can be observed in the CuGA **90–2.2** chromatogram. A shift in the C−O stretching vibration band from 1421 cm^−1^ in GA to 1416 cm^−1^ in CuGA **90–2.2** was observed. Peak shifts at 1305, 1253, and 1022 cm^−1^ in GA spectra to 1309, 1205, and 1050 cm^−1^, respectively in CuGA **90–2.2** spectra all are related to C–O (at phenol) vibration. The shift in the spectral bands suggests a change in the bond length for these groups^26^, which can be attributed to the coordination between GA and Cu.

The TGA result (Fig. 3b) shows that CuGA **90–2.2** was thermally stable up to 219 °C. At higher temperature (220–257 °C), significant weight loss at approximately 43.7% was observed which indicates thermal degradation of the ligand building blocks. A constant weight was observed as the temperature was increased further (> 257 °C). This suggests that no additional breakdown occurred in the sample, and approximately 48.8% weight of Cu-residue remain in metal oxide form. This TGA result is congruent with results on the elemental analysis of the CuGA **90–2.2**; calculated formula Cu_2_C_8_H_6_O_8_ (fw.296): 26.90% C, 1.80% H, and 35.30% O.

N_2_ adsorption/desorption isotherm of CuGA **90–2.2** is shown in Fig. 3c. A hysteresis loop was observed at *P*/*P*_*0*_ = 0.5, and the curve is a type IV isotherm curve which indicates that CuGA **90–2.2** has a mesoporous structure with an H3 hysteresis loop caused by a delay of the desorption process^27–29^. The calculated BET surface area, total pore volume, and average pore diameter of CuGA **90–2.2** are 198.22 m^2^/g, 0.4262 cc/g, and 8.6 nm, respectively which are comparable to the CuGA NMOF reported by Sharma et al. (172 m^2^/g, 0.73 cc/g, and 2.2 nm, respectively)^19^. These results suggest that the newly developed synthetic method at relatively low temperature (90 °C) and in the absence of solvents can be used to produce CuGA MOF with similar characteristics as the DMF-synthesized CuGA NMOF. The SEM image of CuGA (Fig. 3d) shows the irregular granular shaped particles with rough surface morphology. Meanwhile, no specific geometry shape can be observed.

### Effect of pH on the adsorption of dye by CuGA 90–2.2

**Figure 4:**
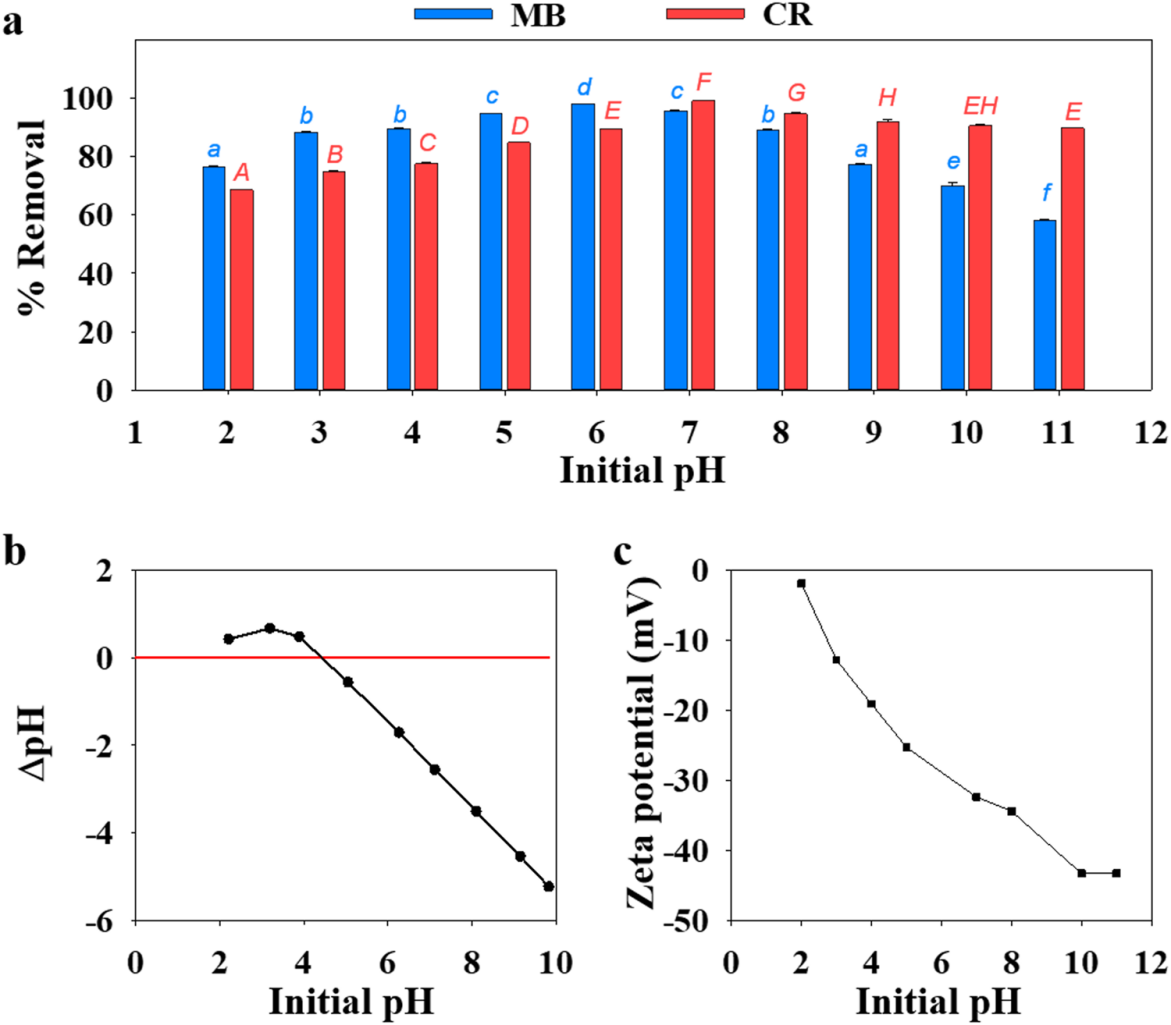
(**a**) Effect of pH on the efficiency of CuGA **90–2.2** for MB (blue bar) and CR (red bar) removal. (**b**) Zeta potential and (**c**) pH_PZC_ measurement of CuGA **90–2.2** at different pH.

One of the critical variables that affect the adsorption process is the initial pH of solution since it affects the surface charge of adsorbent and adsorbate^30^. Figure 4a shows the effect of initial pH value on the adsorption efficiency of CuGA 90–2.2 for MB and CR removal. The percent removal of MB and CR peaked at pH 6 and 7, respectively. This pH-dependent adsorption behavior of MB and CR onto the adsorbent can be explained from the pH_PZC_ and zeta potential of CuGA **90–2.2**, as shown in Fig. 4b. The pH_PZC_ of the CuGA **90–2.2** is about 4.41; thus, at pH 6 and 7, the surface charge of this MOF is negative.

The negative surface charge is also confirmed from the zeta potential value (Fig. 4c). At pH 6, CuGA **90–2.2** has a surface charge that is opposite from the cationic MB, suggesting that their interactions was driven by electrostatic forces^31^. Meanwhile, binding mechanisms such as $\pi$–$\pi$ stacking^32^, might be the contributing force for the interaction of CuGA **90–2.2** and CR since both are negatively charged at pH 7.

### Adsorption isotherm

**Figure 5:**
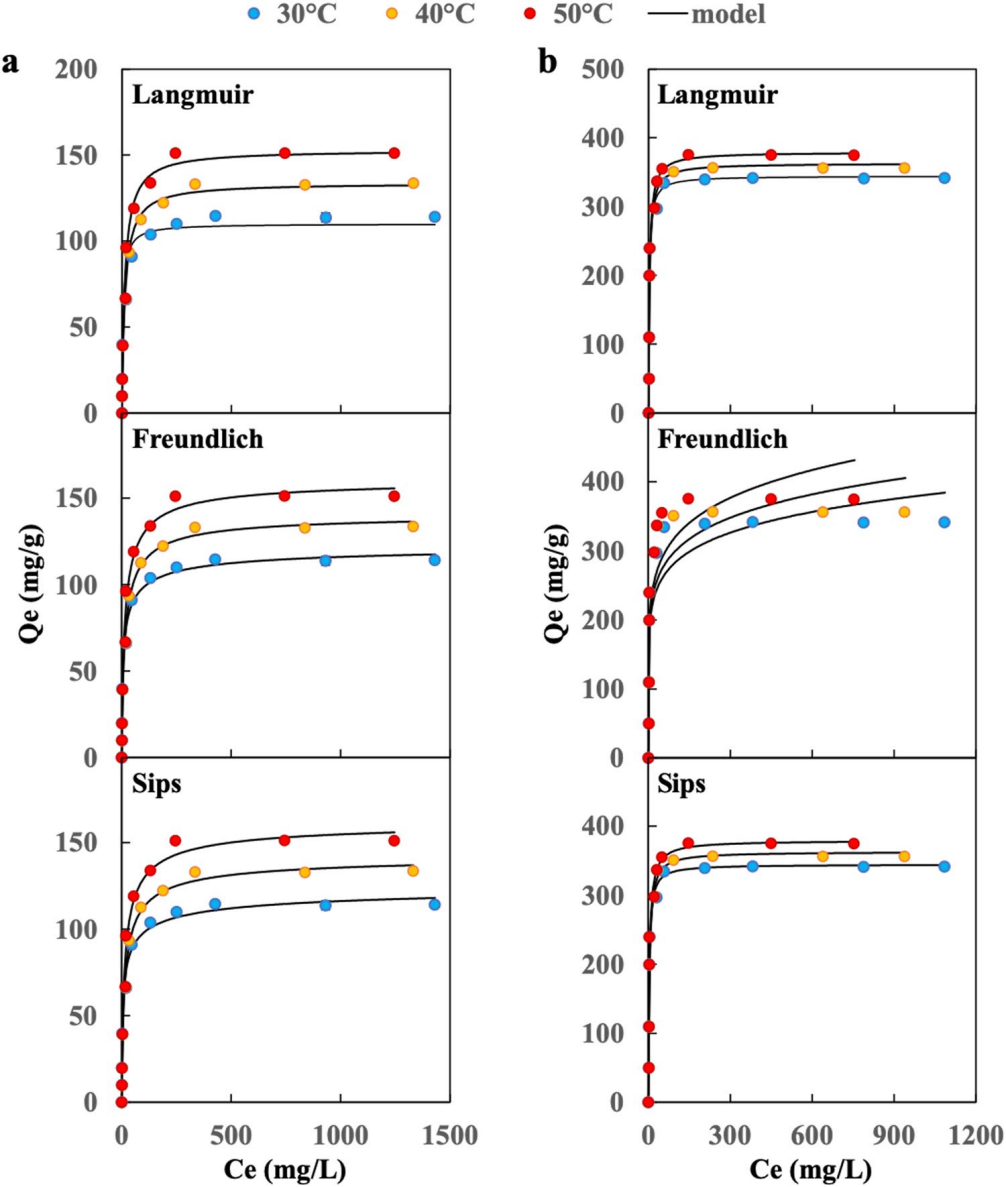
Adsorption isotherm curves of (**a**) MB and (**b**) CR on CuGA **90–2.2** at different temperatures.

The adsorption isotherm gives the relationship between adsorbate and adsorbent in equilibrium condition. The adsorption isotherm curves of MB and CR at 303, 313, and 323 K on the CuGA **90–2.2** are presented in Fig. 5.

Based on the shape of the initial slope, the curves belong to class H-curve with subclass 2; which indicate that the adsorbate molecules have high affinity toward the adsorbent^33^. Furthermore, a long plateau was observed in the curve, which shows that the adsorption of adsorbate reached saturation^34^. 2-Parameter models (i.e., Langmuir, Freundlich) and 3-parameter Sips model were used to fit the adsorption data. The calculated parameters, along with *R*^2^ values, are presented in Table 1. The *R*^2^ values are close to 1 for Langmuir and Sips models, indicating good fittings. Furthermore, the calculated q_max_ of Langmuir and Sips (q_max,L_ and q_max,S_) models also show good agreement with the highest adsorption capacity obtained from experimental data (q_exp_). The dimensionless n parameter of the Sips model can show the heterogeneity tendency of the adsorbent. The value of n parameter in Sips, which is close to 1 implies a homogeneous adsorption process. Although a lower *R*^2^ was obtained for the Freundlich model, the value of n in the Freundlich model can show the favorability of the adsorption. The n parameter of the Freundlich model falls between 2–10 for all systems, indicating that the adsorption is favorable^34^.

**Table 1:** Constants and correlation coefficients of Langmuir, Freundlich and Sips models.

Dye	T (K)	Qe (mg/g)	Langmuir			Freundlich			Sips			
			q_max,L_ (mg/g)	K_L_ (L/g)	R^2^	K_F_	n	R^2^	q_max,S_ (mg/g)	K_S_ (L/mg)	n	R^2^
MB	303	115.60	110.09	0.175	0.946	39.71	6.072	0.906	124.64	0.299	0.551	0.989
	313	133.85	133.78	0.072	0.986	38.49	5.168	0.885	141.25	0.127	0.752	0.995
	323	151.55	152.94	0.072	0.979	44.54	5.196	0.883	161.39	0.131	0.748	0.987
CR	303	343.71	344.54	0.343	0.946	158.06	7.871	0.772	344.54	0.3430	1.000	0.946
	313	357.30	362.92	0.266	0.928	162.21	7.474	0.739	362.92	0.266	1.000	0.928
	323	375.58	379.19	0.257	0.939	163.55	6.835	0.758	379.19	0.257	1.000	0.939

**Figure 6:**
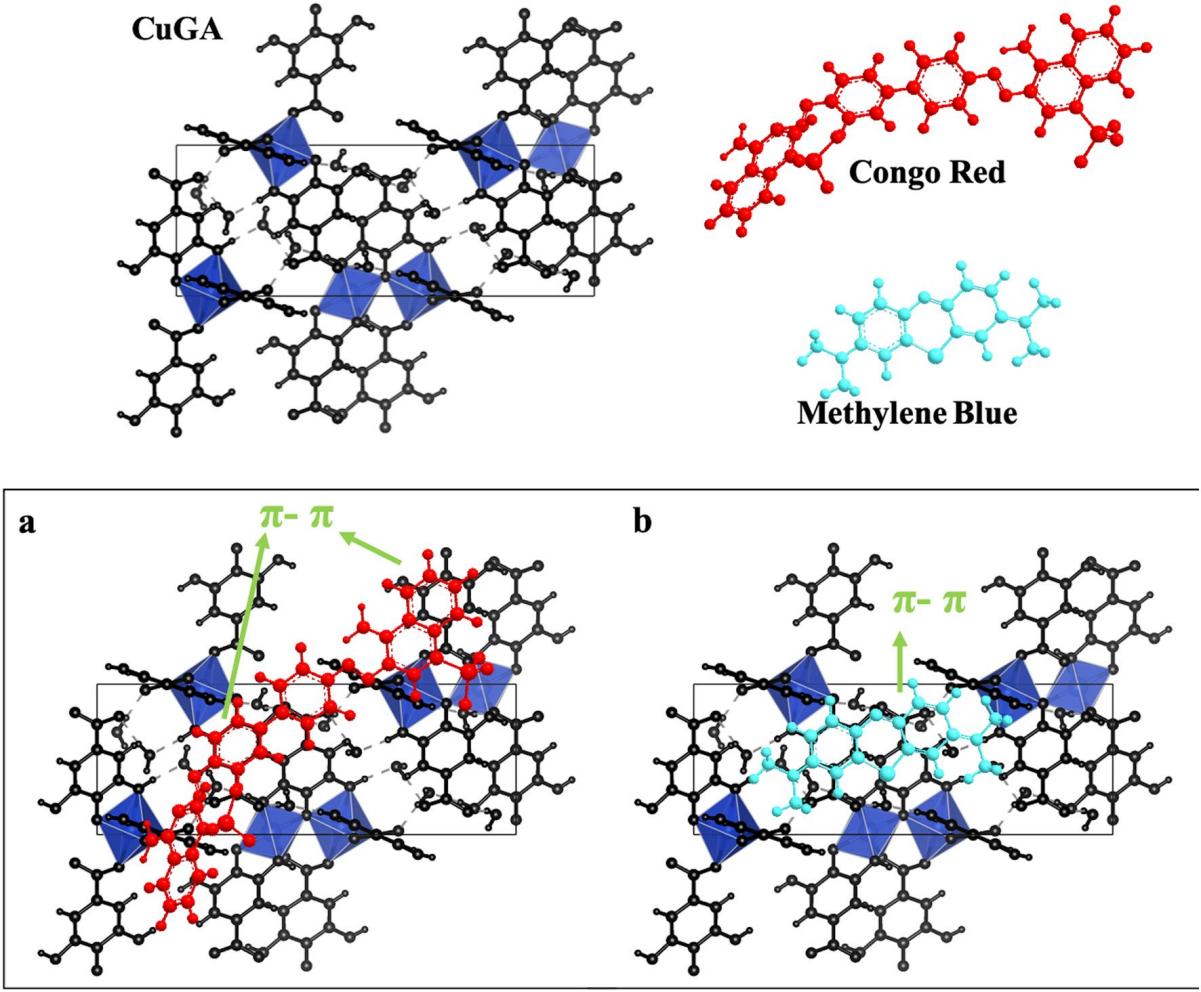
Illustration of possible mechanism of (**a**) CR and (**b**) MB adsorption onto CuGA **90–2.2**.

CuGA **90–2.2** can adsorb a higher amount of CR than MB as indicated by the q_max_ and q_exp_ values. While the adsorbent-adsorbate electrostatic attractions cannot explain this phenomenon, the interaction can be related to the presence of Cu-nodes in CuGA **90–2.2**. In hard-soft acid–base theory, Cu is classified as borderline-soft acid that has better interaction with borderline base^35,36^. Based on its molecular feature, CR possesses sulfonate groups, a borderline base, thus provide better interaction to the Cu-nodes of CuGA **90–2.2**. In addition, the presence of benzene rings in CR and MB structures may contribute to dye adsorption onto CuGA due to the occurrence of π–π interaction (Fig. 6). The greater number of benzene rings in the CR structure facilitates π–π interactions with the CuGA **90–2.2**^32^.

**Figure 7:**
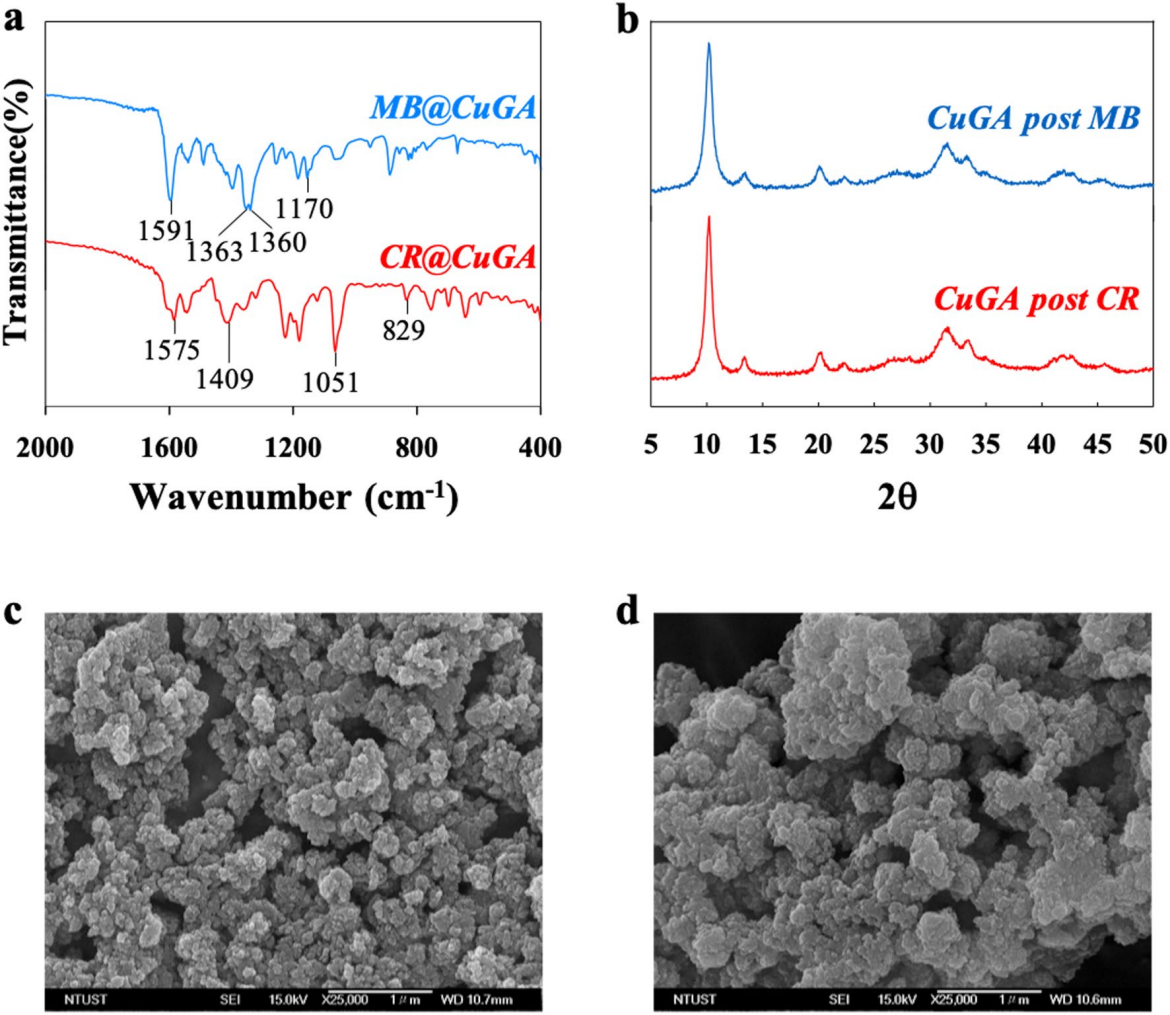
Characterization of post-adsorption CuGA **90–2.2**. (**a**) FTIR spectra of MB-loaded CuGA (MB@CuGA) and CR-loaded CuGA (CR@CuGA). (**b**) XRD crystallography of CuGA after 5 adsorption–desorption cycles. (**c**) and (**d**) SEM image of MB@CuGA (**c**) and CR@CuGA (**d**).

The FTIR, XRD, and SEM analysis were conducted on the post adsorption CuGA **90–2.2**. The FTIR chromatogram of the dye-loaded CuGA **90–2.2** (referred to as MB@CuGA and CR@CuGA) shows the alteration on the spectral shape compared to the spectra of CuGA **90–2.2** before adsorption. Several new absorption bands appear in the spectra of MB@CuGA and CR@CuGA (Fig. 7a), which confirms the adsorbate and adsorbent interactions. In MB@CuGA chromatogram, new peaks appear at 1170, 1360, and 1363–1591 cm^−1^ corresponding to the C=C, aromatic ring, and C–H asymmetric vibration peaks of MB, respectively^37^. On the other hand, the adsorption of CR onto the CuGA surface is indicated by the appearance of CR characteristic peaks at 829, 1051, 1409, and 1575 cm^−1^, which are attributed to the vibration of aromatic rings, S=O stretching vibration, and –N=N– stretching vibration of CR, respectively^38^. SEM images of the MB@CuGA and CR@CuGA was also observed. However, no apparent alteration was observed from the SEM images of the dye-loaded CuGA (Fig. 7c,d).

It worths noting that the synthesized CuGA **90–2.2** has higher adsorption capacity toward MB and CR than the other MOF-adsorbents reported in literatures, as shown in Table 2. The maximum adsorption capacity of CuGA **90–2.2** is 1.4 to 5.7 times higher for MB, and 1.4 to 5.3 times higher for CR than the other reported MOFs. This shows that the synthesized CuGA **90–2.2** can be a promising adsorbent for either anionic or cationic dye adsorption. Furthermore, the aqueous synthesis of CuGA **90–2.2** can be considered as an eco-friendly process.

**Table 2:** Adsorption capacities of various adsorbents for MB and CR removal.

Dye	Adsorbent	Adsorption capacities (mg/g)	Temp (°C)	Ref
MB	CuGA **90–2.2**	124.64	30	This work
	UiO-66	91.00	30	^39^
	MIL-101	21.79	25	^40^
	Fe_3_O_4_@MIL-100	56.15	35	^41^
	NH2-MIL-88B	61.46	25	^42^
CR	CuGA **90–2.2**	344.54	30	This work
	Glu-Cu^2+^ (MOFs)	77.60	37	^43^
	Chitosan/UiO-66	246.21	25	^44^
	ZIF‐8@CoFe2O4	64.48	30	^45^
	Fe_3_O_4_/Bi_2_S_3_ MSs	92.24	55	^46^

### Adsorption thermodynamics

The thermodynamic parameters, including the changes in enthalpy (ΔH), free energy (ΔG), and entropy (ΔS), in the adsorption of MB and CR onto CuGA **90–2.2** were determined. According to the linear relationships, the parameter ΔH and ΔS can be obtained as the slope and intercept of 1/T versus ln (K_C_) plot which obtained based on the following equations:

$$\ln{K}_{C}={\frac{\Delta \text{S}}{R}}-{\frac{\Delta \text{H}}{RT}}$$

where R is the universal gas constant (8.314 J/mol K), T is the temperature (K), and *K*_C_ is the dimensionless distribution coefficient for the adsorption. The value of *K*_C_ can be calculated as the ratio between the equilibrium concentration on the solid phase (*C*_*a*_, mg/L) and in the solution (*C*_*e*_, mg/L)^47^. ΔG was calculated using the equation below:

$$\Delta G= -\mathrm{RT ln }({K}_{c})$$

The calculated values of ΔH, ΔS, and ΔG are given in Table 3. The negative ΔH of the adsorption systems denote that the heat was released during the process and that the adsorption occurred exothermically^48^. The negative ΔS implies the decrease in the degree of freedom of the molecules due to the fact that the bonded adsorbate molecules have less freedom of movement than when they are free in solution. Compared to the MB system, the less negative of ΔS value for the CR system can be attributed to the softer binding potential in this system^49^. The negative ΔG values of MB and CR adsorption indicate the thermodynamic favorability and spontaneous nature of the adsorption process^50^.

**Table 3:** Thermodynamics parameters of MB and CR adsorption on CuGA **90–2.2**.

Dye	∆H (kJ/mol)	∆S (J/mol)	∆G (kJ/mol)		
			303 K	313 K	323 K
MB	− 19.3517	− 50.2856	− 4.2442	− 3.3373	− 3.2561
CR	− 10.5147	− 16.0269	− 5.6700	− 5.4106	− 5.3851

### Reusability

**Figure 8:**
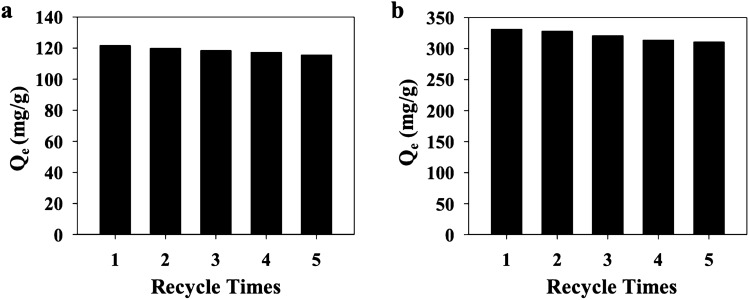
Effect of recycle number of Cu-GA MOF on the **(a)** MB and **(b)** CR adsorption capacity.

Adsorbents with high reusability can benefit from the aspects of process-cost savings, environmental friendliness, and practicality^51^. Ethanol was employed as the desorption agent for the regeneration of CuGA. The CuGA showed excellent recyclability, retaining high adsorption efficiency (> 90%) after five adsorption–desorption cycles (Fig. 8). After fifth adsorption–desorption cycles, the efficiency of CuGA is 94.96% and 93.82% for MB and CR, respectively—with the corresponding adsorption capacity for MB and CR removal of 115.51 mg/g and 310.67 mg/g, respectively. To confirm the stability of CuGA **90–2.2** after 5 adsorption–desorption cycles, the PXRD patterns of the CuGA were recorded. As shown in Fig. 7b, there is no significant change in the PXRD pattern of the adsorbent before and after 5 cycles of dye removal.

## Conclusion

An aqueous synthesis method of CuGA MOF has been developed to replace the conventional synthesis method. Two important conditions in the synthesis that affect the successful formation of CuGA MOF are the synthesis temperature and the molar ratio of NaOH to GA. A proper amount of NaOH addition is important in promoting the formation of CuGA MOF while preventing metal oxide formation. A synthesis temperature of 90 °C was found to facilitate the formation of CuGA with stable coordination. Synthesis temperature also profoundly affected the adsorption ability of the resulting CuGA MOF for dye removal. Compared to other extensively studied MOFs, the aqueous-synthesized CuGA MOF possesses higher adsorption capacity for MB and CR. The CuGA MOF showed high reusability with adsorption efficiency decreased less than 10% after 5 adsorption–desorption cycles.

## Materials and methods

### Materials

Copper (II) chloride dihydrate (CuCl_2_.2H_2_O, CAS 10125-13-0, ≥ 99% purity) and gallic acid (C_7_H_6_O_5_, CAS 149-91-7, ≥ 97.5% purity) were obtained from Sigma-Aldrich (St Louis, MO). Sodium hydroxide (NaOH, CAS 1310-73-2, ≥ 97% purity) was obtained from Fisher Scientific (Leicestershire, UK). CR (C.I. 22,120, CAS 573-58-0) was supplied by Alfa Aesar (China). MB (C.I. 52,015, CAS 7220-79-3, ≥ 95% purity) and hydrochloric acid (HCl, CAS 7647-01-0, 37%) were purchased from Acros Organics (New Jersey, USA). All chemicals were used directly as obtained without any further purification.

### Synthesis of CuGA MOF

For CuGA MOF synthesis, 2.5 mmol (0.425 g) of GA was dissolved in 5 mL NaOH solution at certain molar concentration (Table 4). Into the GA solution, 2.5 mmol (0.426 g) CuCl_2_.2H_2_O solution in 5 ml DI water was added dropwise. This mixture was then stirred for 2 h at constant temperature. The dark brown solids obtained from the reaction were collected then rinsed with DI water 5 times. Finally, the solids were freeze-dried using a Labconco freeze dryer for 24 h. The as-synthesized solids were referred to as CuGA T-***X*** (T refers to the reaction temperature; ***X*** refers to the molar ratio of NaOH to GA, as listed in Table 4).

**Table 4:** Conditions used to synthesize CuGA MOF.

Sample name	Concentration (M)			Temp	pH_measured_
	CuCl_2_.2H_2_O	GA	NaOH		
**First optimization step**					
60–1.1	0.5	0.5	0.55	60	3.71 ± 0.13
60–2.2	0.5	0.5	1.10	60	4.64 ± 0.37
60–3.3	0.5	0.5	1.65	60	7.30 ± 0.05
60–4.4	0.5	0.5	2.42	60	8.53 ± 0.13
**Second optimization step**					
30–2.2	0.5	0.5	1.10	30	4.51 ± 0.08
90–2.2	0.5	0.5	1.10	90	4.13 ± 0.14

### Characterization of CuGA MOF

Elemental analysis of the complex was carried out using a Thermo Flash 2000 CHNS/O Analyzers. The crystallinity pattern was determined by using a Powder X-Ray Diffraction (PXRD, Bruker D2 PHASER XE-T XRD) operated at 30 kV; 10 mA using Cu-Kα radiation (λ = 0.154060 nm) at 3° PSD opening, 0.5 times per step and 2Theta from 5° to 60°. The surface functional group analysis was performed by using a Fourier-Transform Infrared Spectroscopy (FTIR, SHIMADZU Tracer-100); the analysis was carried out in the range of 400–4000 cm^−1^ wavenumbers and KBr pellets were applied as the background. Thermogravimetric analysis (TGA, TA instruments/TGA 550) was carried out at temperature range 30–650 °C with a heating rate of 10 °C/min. The isotherms of N_2_ adsorption–desorption were measured at 77 K using a BELSORP-max analyzer. The samples were degassed for 4 h at 383 K before analyzing. The specific surface area was calculated from the adsorption branch using the Brunauer–Emmett–Teller (BET) model. The pH_PZC_ and zeta potential measurement were done according to the previous report^34,52^.

### Adsorption experiment

CuGA MOF powder (20 mg) was mixed with 2 mL dye solution. CR and MB were chosen as the model adsorbate in this work at a respective initial concentration of 500–4500 mg/L and 10–2000 mg/L. The adsorption was carried out in a shaking incubator for 24 h, at 200 rpm, under a constant temperature of 303, 313, and 323 K. The remaining dyes were then separated from CuGA MOFs by centrifugation (Smart 15 Plus Micro Centrifuge) at 15,000 rpm (21,055 g) for 20 min. A UV–visible spectrophotometer (Shimadzu UV 2600) was used to determine the concentration of the remaining CR or MB left in the supernatant (${C}_{e}$). The adsorption isotherm data were then plotted as ${C}_{e}$ vs ${Q}_{e}$. ${Q}_{e}$ were calculated by the following equation:

$${Q}_{e}=\frac{({C}_{0}-{C}_{e})}{W} V$$

where ${Q}_{e}$ (mg/g) is the equilibrium amount of adsorbate adsorbed, ${C}_{0}$ and ${C}_{e}$ (mg/L) represents the initial and final (post-adsorption) concentrations of dye, respectively, $V$ (L) is the volume of dye solution, and $W$ (g) is the weight of CuGA MOF.

### Adsorption modeling

SigmaPlot 12.5, a Systat software, was used for adsorption data modeling and fitting. The models used include 2-parameters model (i.e., Langmuir, Freundlich) and 3-parameters Sips models. The Langmuir model can be expressed as:

$$Qe=\frac{{Q}_{max}{K}_{L}{C}_{e}}{{1+K}_{L}{C}_{e}}$$

where ${K}_{L}$ (L/g) is the Langmuir affinity constant related to the energy of adsorption, and ${Q}_{max}$ (mg/g) is the maximum adsorption capacity of the CuGA MOF with assumption of monolayer surface coverage^53^.

Freundlich isotherm model was used to describe the multilayer adsorption with the interaction of adsorbent and adsorbed molecules. Freundlich model is represented by the following equation:

$${Q}_{e}={{{K}_{F} C}_{e}}^{1/n}$$

where ${K}_{F}$((mg/g)(L/mg)^−1/n^) is the Freundlich constant related to the adsorption capacity of the adsorbent, and *n* (dimensionless) is the heterogeneity and adsorption intensity of adsorbent^54^.

Sips isotherm model is a combined form of Langmuir and Freundlich expressions designed for predicting the heterogeneous adsorption systems while evading the limitation of rising adsorbate concentration. At low adsorbate concentrations, it reduces to Freundlich isotherm; and it predicts monolayer adsorption, a characteristic of the Langmuir isotherm, at high adsorbate concentrations. Sips isotherm model is represented by following equation:

$$Qe=\frac{{Q}_{ms} {{{K}_{S} C}_{e}}^{\beta}}{1+Ks{{ C}_{e}}^{\beta}}$$

where ${Q}_{ms}$(mg/g) is the Sips maximum adsorption capacity of the adsorbent, Ks (L mg^−1^) is the Sips equilibrium constant, and β is the Sips model exponent, which can also be employed to describe the system’s heterogeneity when is between 0 and 1. When β = 1, the Sips equation reduces to the Langmuir equation which implies a homogeneous adsorption process^34^.

## References

[1] Hu J (2014). Enhanced adsorptive removal of hazardous anionic dye “congo red” by a Ni/Cu mixed-component metal–organic porous material. RSC Adv..

[2] Gupta VK, Jain R, Nayak A, Agarwal S, Shrivastava M (2011). Removal of the hazardous dye—Tartrazine by photodegradation on titanium dioxide surface. Mater. Sci. Eng., C.

[3] Liu L (2015). Simultaneous removal of cationic and anionic dyes from environmental water using montmorillonite-pillared graphene oxide. J. Chem. Eng. Data.

[4] Nodehi R, Shayesteh H, Kelishami AR (2020). Enhanced adsorption of congo red using cationic surfactant functionalized zeolite particles. Microchem. J..

[5] Khaniabadi YO (2017). Adsorption of congo red dye from aqueous solutions by montmorillonite as a low-cost adsorbent. Int. J. Chem. React. Eng..

[6] Vandevivere PC, Bianchi R, Verstraete W (1998). Treatment and reuse of wastewater from the textile wet-processing industry: Review of emerging technologies. J. Chem. Technol. Biotechnol..

[7] Ariyanti D, Maillot M, Gao W (2018). Photo-assisted degradation of dyes in a binary system using TiO_2_ under simulated solar radiation. J. Environ. Chem. Eng..

[8] Vandevivere PC, Bianchi R, Verstraete WJJ (1998). Biotechnology: International research in process, E & technology, C treatment and reuse of wastewater from the textile wet-processing industry. Rev. Emerg. Technol..

[9] Jia J (2019). Extremely hydrophobic POPs to access highly porous storage media and capturing agent for organic vapors. Chemistry.

[10] Dissegna S (2017). Using water adsorption measurements to access the chemistry of defects in the metal–organic framework UiO-66. CrystEngComm.

[11] Haque E, Jun JW, Jhung SH (2011). Adsorptive removal of methyl orange and methylene blue from aqueous solution with a metal-organic framework material, iron terephthalate (MOF-235). J. Hazard. Mater..

[12] Khan NA, Jung BK, Hasan Z, Jhung SH (2015). Adsorption and removal of phthalic acid and diethyl phthalate from water with zeolitic imidazolate and metal–organic frameworks. J. Hazard. Mater..

[13] Mirsoleimani-azizi SM, Setoodeh P, Zeinali S, Rahimpour MR (2018). Tetracycline antibiotic removal from aqueous solutions by MOF-5: Adsorption isotherm, kinetic and thermodynamic studies. J. Environ. Chem. Eng..

[14] Jamali A, Tehrani AA, Shemirani F, Morsali A (2016). Lanthanide metal–organic frameworks as selective microporous materials for adsorption of heavy metal ions. Dalton Trans..

[15] Abbasi AR, Karimi M, Daasbjerg K (2017). Efficient removal of crystal violet and methylene blue from wastewater by ultrasound nanoparticles Cu-MOF in comparison with mechanosynthesis method. Ultrason. Sonochem..

[16] Badhani B, Sharma N, Kakkar R (2015). Gallic acid: A versatile antioxidant with promising therapeutic and industrial applications. RSC Adv..

[17] Kahkeshani N (2019). Pharmacological effects of gallic acid in health and diseases: A mechanistic review. Iran. J. Basic Med. Sci..

[18] Dhaka S (2019). Metal–organic frameworks (MOFs) for the removal of emerging contaminants from aquatic environments. Coord. Chem. Rev..

[19] Sharma S, Mittal D, Verma AK, Roy I (2019). Copper-gallic acid nanoscale metal-organic framework for combined drug delivery and photodynamic therapy. ACS Appl. Bio Mater..

[20] Kim TH, Kim SG (2011). Clinical outcomes of occupational exposure to N, N-dimethylformamide: Perspectives from experimental toxicology. Saf. Health Work.

[21] Angkawijaya AE, Fazary AE, Ismadji S, Ju Y-H (2012). Cu(II), Co(II), and Ni(II)–antioxidative phenolate-glycine peptide systems: An insight into its equilibrium solution study. J. Chem. Eng. Data.

[22] Fazary AE (2011). Complex formation between ferric(III), chromium(III), and cupric(II) metal ions and (O, N) and (O, O) donor ligands with biological relevance in aqueous solution. J. Solution Chem..

[23] McGuire CV, Forgan RS (2015). The surface chemistry of metal–organic frameworks. Chem. Commun..

[24] Chen K, Xue D (2012). pH-assisted crystallization of Cu_2_O: Chemical reactions control the evolution from nanowires to polyhedra. CrystEngComm.

[25] Wolfenden BS, Willson RL (1982). Radical-cations as reference chromogens in kinetic studies of ono-electron transfer reactions: Pulse radiolysis studies of 2,2′-azinobis-(3-ethylbenzthiazoline-6-sulphonate). J. Chem. Soc. Perkin Trans..

[26] Haas KL, Franz KJ (2009). Application of metal coordination chemistry to explore and manipulate cell biology. Chem. Rev..

[27] Sing KSW, Williams RT (2004). Physisorption hysteresis loops and the characterization of nanoporous materials. Adsorpt. Sci. Technol..

[28] Cychosz KA, Guillet-Nicolas R, García-Martínez J, Thommes M (2017). Recent advances in the textural characterization of hierarchically structured nanoporous materials. Chem. Soc. Rev..

[29] Ling S, Walton RI, Slater B (2015). Theoretical study of conformational disorder and selective adsorption of small organic molecules in the flexible metal-organic framework material MIL-53-Fe. Mol. Simul..

[30] Ustunol IB, Gonzalez-Pech NI, Grassian VH (2019). pH-dependent adsorption of α-amino acids, lysine, glutamic acid, serine and glycine, on TiO_2_ nanoparticle surfaces. J. Colloid Interface Sci..

[31] Robati D, Bagheriyan S, Rajabi M, Moradi O, Peyghan AA (2016). Effect of electrostatic interaction on the methylene blue and methyl orange adsorption by the pristine and functionalized carbon nanotubes. Phys. E.

[32] Jagusiak A, Piekarska B, Chłopaś K, Bielańska E, Irena R, Leszek K (2018). Self-Assembled Molecules—New Kind of Protein Ligands: Supramolecular Ligands.

[33] Piccin JS, Cadaval TRSA, de Pinto LAA, Dotto GL, Adrián BP, Didilia Ileana MC, Hilda Elizabeth RÁ (2017). Adsorption Processes for Water Treatment and Purification.

[34] Angkawijaya AE (2020). Studies on the performance of bentonite and its composite as phosphate adsorbent and phosphate supplementation for plant. J. Hazard. Mater..

[35] Pearson RG (1968). Hard and soft acids and bases, HSAB, part 1: Fundamental principles. J. Chem. Educ..

[36] Pearson RG (1963). Hard and soft acids and bases. J. Am. Chem. Soc..

[37] Pradhan AC, Paul A, Rao GR (2017). Sol-gel-cum-hydrothermal synthesis of mesoporous Co-Fe@Al2O3−MCM-41 for methylene blue remediation. J. Chem. Sci..

[38] Zhang X, Zhang Y, Wang D, Qu F (2014). Investigation of adsorption behavior of Cu_2_O submicro-octahedra towards congo red. J. Nanomater..

[39] Mohammadi A (2017). Metal-organic framework Uio-66 for adsorption of methylene blue dye from aqueous solutions. Int. J. Environ. Sci. Technol..

[40] Shen T, Luo J, Zhang S, Luo X (2015). Hierarchically mesostructured MIL-101 metal–organic frameworks with different mineralizing agents for adsorptive removal of methyl orange and methylene blue from aqueous solution. J. Environ. Chem. Eng..

[41] Shao Y (2016). Magnetic responsive metal–organic frameworks nanosphere with core–shell structure for highly efficient removal of methylene blue. Chem. Eng. J..

[42] He J, Zhang Y, Zhang X, Huang Y (2018). Highly efficient Fenton and enzyme-mimetic activities of NH 2-MIL-88B (Fe) metal organic framework for methylene blue degradation. Sci. Rep..

[43] Pu F, Liu X, Xu B, Ren J, Qu X (2012). Miniaturization of metal-biomolecule frameworks based on stereoselective self-assembly and potential application in water treatment and as antibacterial agents. Chem. A Eur. J..

[44] Wen L (2020). Ice-templated porous polymer/UiO-66 monolith for Congo Red adsorptive removal. Arab. J. Chem..

[45] Xu Y (2016). Rapid magnetic solid-phase extraction of congo red and basic red 2 from aqueous solution by ZIF-8@ CoFe2O4 hybrid composites. J. Sep. Sci..

[46] Zhu H (2017). Magnetically recyclable Fe3O4/Bi2S3 microspheres for effective removal of Congo red dye by simultaneous adsorption and photocatalytic regeneration. Sep. Purif. Technol..

[47] Ahmaruzzaman M, Laxmi Gayatri S (2010). Batch adsorption of 4-nitrophenol by acid activated jute stick char: Equilibrium, kinetic and thermodynamic studies. Chem. Eng. J..

[48] Anfar Z (2019). Combined methane energy recovery and toxic dye removal by porous carbon derived from anaerobically modified digestate. ACS Omega.

[49] Ben-Tal N, Honig B, Bagdassarian CK, Ben-Shaul A (2000). Association entropy in adsorption processes. Biophys. J ..

[50] Chabani M, Amrane A, Bensmaili A (2006). Kinetic modelling of the adsorption of nitrates by ion exchange resin. Chem. Eng. J..

[51] Vakili M (2019). Regeneration of chitosan-based adsorbents used in heavy metal adsorption: A review. Sep. Purif. Technol..

[52] Ibrahim AH (2019). Tuning the chemical environment within the UiO-66-NH2 nanocages for charge-dependent contaminant uptake and selectivity. Inorg. Chem..

[53] Tsang DC (2007). Activated carbon produced from waste wood pallets: Adsorption of three classes of dyes. Water Air Soil Pollut..

[54] Chen L (2017). Environmental-friendly montmorillonite-biochar composites: Facile production and tunable adsorption-release of ammonium and phosphate. J. Clean. Prod..

